# Unspecific DNA recombination in AdipoqCre-ER^T2^ – mediated knockout approaches in transgenic mice is sex-, age- and genotype-dependent

**DOI:** 10.1080/21623945.2019.1701394

**Published:** 2019-12-16

**Authors:** Andreas Lindhorst, Ingo Bechmann, Martin Gericke

**Affiliations:** a Institute of Anatomy and Cell Biology, Martin-Luther-University Halle-Wittenberg, Halle (Saale), Germany; b Institute of Anatomy, Leipzig University, Leipzig, Germany

**Keywords:** Cre recombinase, Cre leakage, Cre/loxP, diabetes, adipose tissue, adiponectin promoter

## Abstract

Due to the epidemic rise of obesity prevalence, adipose tissue (AT) research is of major interest. Our aim was to study specificity of the most-common Cre/loxP approach for inducible gene manipulation of AT in mice (AdipoqCre-ER^T2^). We used mice with tamoxifen-sensitive Cre recombinase controlled by the adiponectin promoter (AdipoqCre-ER^T2^), which were crossed to a tdTomato reporter mouse to visualize the site of recombination on a single-cell resolution. Albeit tamoxifen induced tdTomato expression in this model, also non-stimulated background recombination (‘Cre leakage’) was detected in AT of untreated Adipoq-CreER^T2^xTDTO mice *in vivo*. Quantification of Cre leakage revealed age, sex and genotype as factors impacting on non-induced Cre recombination.

## Introduction

Transgenic mouse models have become a very important tool for researches in almost every field. One very successful method for creating cell type-specific, somatic knockouts is the Cre/loxP system. The Cre recombinase catalyzes cite specific DNA recombination between so-called loxP sites [1]. Using tissue-specific Cre drivers limits knockouts to target cell types rather than full-body knockouts [2]. Introducing a fusion protein of the Cre recombinase to a mutated ligand-binding domain of the human oestrogen receptor also facilitated inducible knockouts [3]. The Cre-ER^T^ fusion protein cannot access the DNA until 4-hydroxy-tamoxifen (TAM) induces the translocation into the nucleus enabling DNA recombination. A second generation of fusion proteins, Cre-ER^T2^, is 10x more sensitive to TAM, but cannot be activated by natural ligands of the estragon receptor [4].

Adipose tissue (AT) not only serves as storage site for fatty acids but modulates metabolism by releasing peptide hormones and other proteins [5]. Pathological expansion of AT increases the risk of numerous diseases, including type 2 diabetes, coronary heart disease, and hypertension [6]. Therefore, studying adipocyte function became a main subject in diabetes research. A well established and widely used method for targeting genes specifically in adipocytes is the Cre/loxP system controlled by the aP2 promotor [7]. However, recent studies suggest using the adiponectin promotor (Adipoq) for better results due to superior specificity [8]. Of note, the adiponectin promoter, in contrast to the aP2 promoter, has no known ectopic expression in other cells than adipocytes. Hence, adiponectin-driven Cre expression allows for a homogenous and specific recombination in adipocytes of several AT depots, including subcutaneous, perigonadal, retroperitoneal, mesenteric and brown AT [8]. In addition, the transgenic AdipoqCreER^T2^-mouse line, developed by Sassmann and colleagues [9], is a powerful tool to manipulate gene expression in adipocytes of living mice in a time-dependent manner following TAM administration [10]. However, TAM administration in living mice has also been suggested to affect adipocyte biology [11] and may even induce adipocyte death *in vivo* and *in vitro* [12,13]. Therefore, we aimed at establishing a Cre/loxP-based *ex vivo* system to visualize adipocytes and study the site of DNA recombination in real time using a live-imaging approach as described previously [14]. Further, we wanted to analyse the impact of TAM on mature adipocytes in living AT explants. We crossed AdipoqCreER^T2^-mice [9] to mice carrying a Cre-dependent reporter construct on the Rosa26 locus [15]. Thus, Cre-catalysed DNA recombination leads to excision of a stop cassette with subsequent expression of the red fluorescent protein tdTomato (TDTO) in adult adipocytes (‘Adipoq-CreER^T2^xTDTO mice’).

However, a known issue of using the Cre-ER^T2^ system is non-induced recombinase activity (so-called ‘leakage’). Regardless of the used promotor, many Cre-ER^T2^ lines have shown Cre-mediated DNA recombination in absence of TAM [16]. While characterizing the inducibility of TDTO expression in our AT culture model of Adipoq-CreER^T2^xTDTO mice, we observed the previously described Cre leakage without TAM administration. Therefore, we aimed at analysing non-induced DNA recombination in Adipoq-CreER^T2^xTDTO mice in a more detailed manner using mice of different genotype, sex and age. Importantly, we found that leakage of Cre-ER^T2^ recombination in Adipoq-CreER^T2^ mice is sex-, age- and genotype-dependent.

## Methods

### Mice

Animal experiments followed the ‘Principles of laboratory animal care’ (NIH publication no. 85e23, revised 1985) as well as specific national laws approved by the local authorities of the state of Saxony, Germany. All mice were housed in pathogen-free facilities in groups of three to five at 22 ± 2°C on a 12-h light/dark cycle. All animals had free access to water and were fed with standard chow (Sniff GmbH, Soest, Germany). To visualize the site of DNA recombination in the AdipoqCreER^T2^-mouse model [9], we crossed AdipoqCreER^T2^-mice, with a specific CreER^T2^ expression in adipocytes under the adiponectin promoter, to TDTO reporter mice [15], which harbour a tdTomato gene silenced by an upstream located and loxP site flanked Stop codon. Therefore, Cre-mediated excision of the Stop codon upstream of the reporter gene in the offspring generation leads to expression of the red fluorescent protein tdTomato in cells with successful Cre-mediated recombination. Mice were maintained on a C57BL6 background. In regard to the global health status of the animals (e.g. weight, reproduction rate and microbiological status), transgenic mice were indistinguishable from C57BL6 wild-type mice. Genotyping was performed as described previously [11,17]. Allocation to experimental groups were based on genotyping.

### Adipose tissue explant culture

We used young, male and heterozygote Adipoq-CreER^T2^xTDTO reporter mice for visualization of recombination in live AT after tamoxifen treatment using an established tissue culture model [14]. Briefly, after sacrifice, the rostral part of the epididymal white adipose tissue (EWAT) was dissected under sterile conditions and transferred to a PBS-filled culture dish (Life Technologies, Darmstadt, Germany). Subsequently, AT was cut into small tissue blocks of <1 mm^3^ using a sterile razor blade. Next, AT explants were buffer rinsed and five explants per well moved to a six-well plate filled with 1 ml of RPMI cell culture medium supplemented with 10% foetal bovine serum (Sigma-Aldrich), a 1% insulin-transferrin-selenium mixture [1.0 mg/ml bovine insulin, 0.55 mg/ml human transferrin (iron-free), and 0.5 µg/ml sodium selenite; Sigma-Aldrich] and antibiotics (100 U/ml penicillin and streptomycin; Sigma-Aldrich). Importantly, AT explants were stabilized at the bottom of the wells by placing a sterile cell culture insert (Millipore, Billerica, MA) on top of the tissue. AT explants were cultured at 5% CO_2_, 21% O_2_ and 37°C. Live imaging was performed using an inverted FV300 confocal microscope (Olympus, Hamburg, Germany). Acquisition of images was started 1 h after finishing cultivation process, followed by adding 1 µM, 10 µM TAM or vehicle solution (1 mM Stock in ethanol; Sigma-Aldrich). To cover depth of the AT explants, 150 µm thick stacks were collected with 10 µm distance between individual slices.

### Whole mount staining

Whole mounts of AT from reporter mice of different age, sex and genotypes were used for quantifying TDTO expressing adipocytes. Epididymal AT was immediately fixated after sacrifice for 20 min in zinc formalin (Polysciences), washed in PBS, cut into small pieces (<1 mm^3^) and stained for 1 h in HCS LipidTOX™ Deep Red Neutral Lipid Stain (1% in PBS; ThermoFischer). After washing three times in PBS, AT pieces were transferred into cavities of microscope slides and mounted using Fluorescence Mounting Medium (Dako). Image acquisition was performed using an inverted confocal microscope (FV1000 Olympus, Hamburg, Germany) using appropriate, narrow band-pass filters.

### Quantification and statistical analysis

For each reporter mouse of different age, sex and genotypes at least 10 images from randomly chosen positions of the mounted AT were taken and the total number of adipocytes and the number of TDTO expressing adipocytes counted in each image. Statistical analysis was conducted using the GraphPad Prism software (GraphPad Software 8.0, La Jolla, CA, USA), statistical evidence was evaluated by Mann–Whitney U-Test. A p-value of <0.05 was considered statistically significant.

## Results

**Figure 1.**
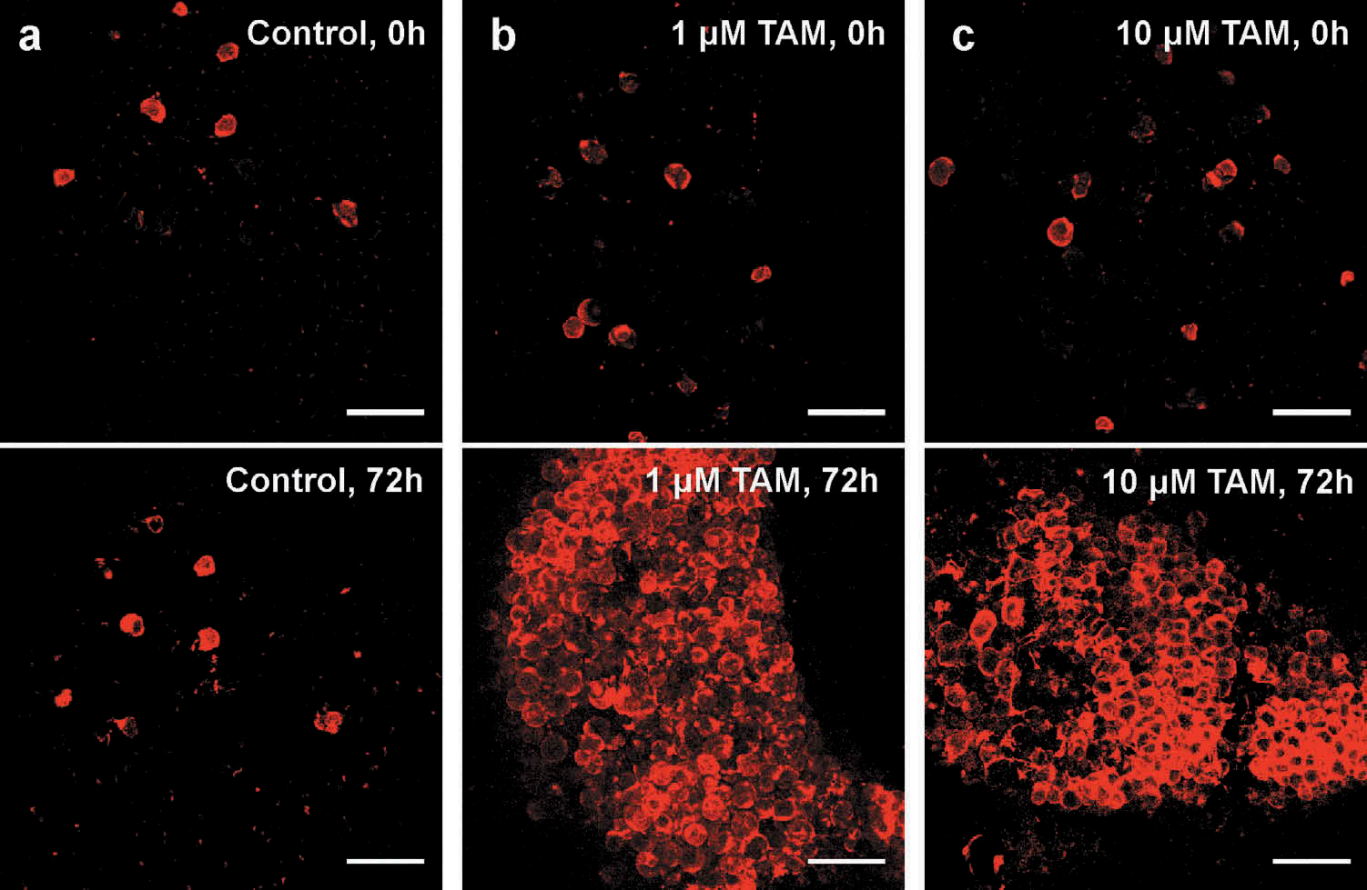
Inducible TDTO Expression Induction of TDTO expression in AT explants derived from young, heterozygous, male Adipoq-CreERT^2^xTDTO reporter mice (b, c) and non-treated controls (a). TDTO fluorescence was detected before and 72 h after TAM induction. Scale bar represents 250 µm

AT explants were cultivated using an established organotypic tissue culture model [14]. After administration of TAM, an increasing number in TDTO expressing adipocytes could be detected 24 h after induction (data not shown) plateauing after 72 h (Figure 1(b,c)). A concentration of 10 µM TAM induced TDTO expression in almost all adipocytes after 72 h. Same result is reached by stimulating with only 1µM TAM, whereas no increase in the number of TDTO^+^ adipocytes could be detected in control explants without TAM (Figure 1(a)). Importantly, TAM did not induce adipocyte death *ex vivo*, as suggested previously [13]. Further, no ectopic TDTO expression was noted in other cells than adipocytes. Nevertheless, in every AT explant, a substantial number of adipocytes already exhibited TDTO expression without TAM administration.

**Figure 2.**
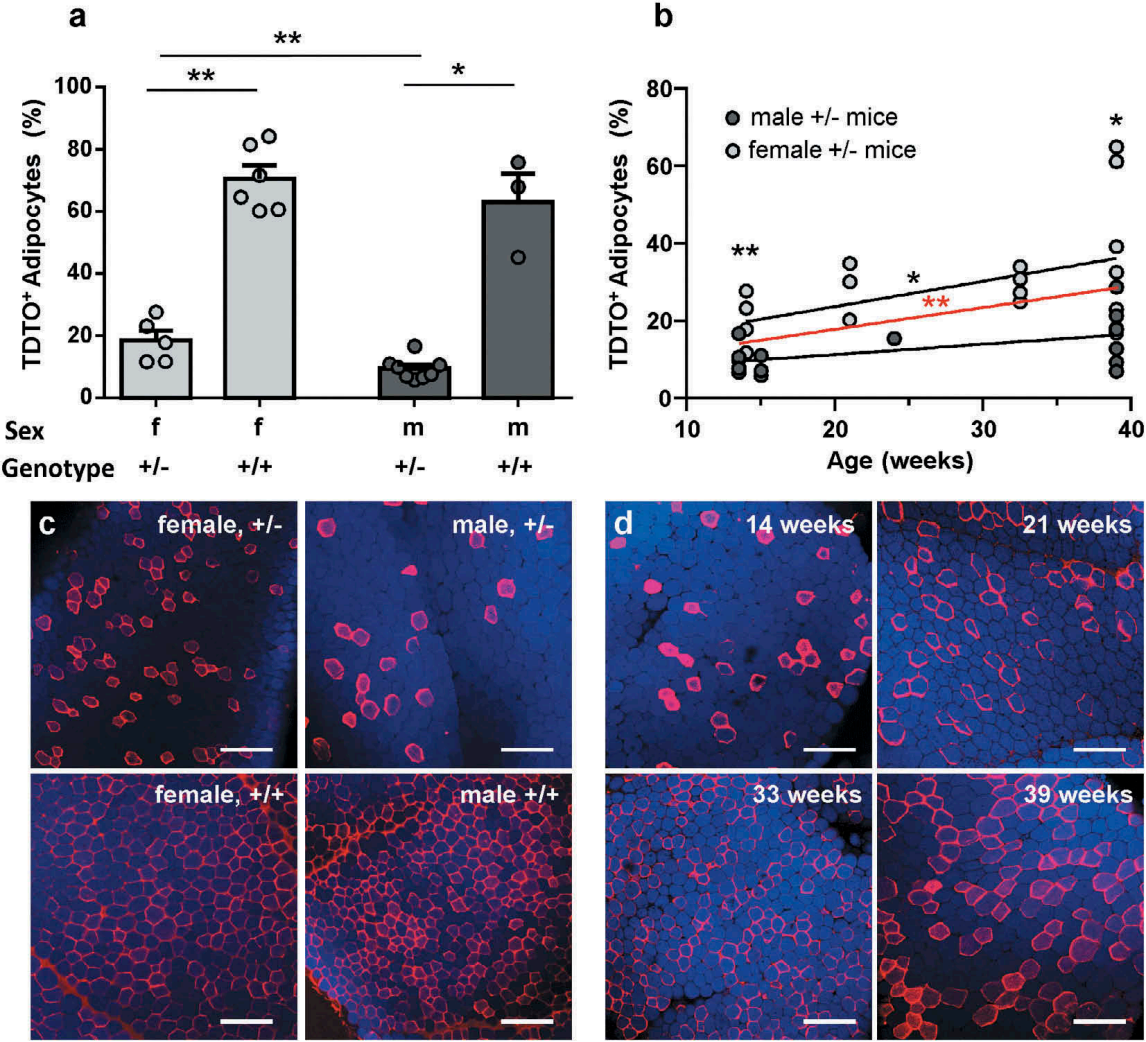
Non-induced TDTO expression in AT from Adipoq-CreER^T2^xTDTO mice Non-induced TDTO expression is studied in Adipoq-CreER^T2^xTDTO reporter mice of different sex, genotype and age. A, Bar graph summarizes non-stimulated TDTO expression in young (<15 weeks) Adipoq-CreER^T2^xTDTO mice of different sex and genotype. B, Linear regression is shown for non-induced TDTO expression in correlation to age of male and female mice. The red line represents the overall regression independent of sex. C and d, representative images for homozygous and heterozygous, female and male mice (c) and for heterozygous, female mice of different ages (d). TDTO fluorescence red, Lipidtox^TM^ fluorescence blue. Data are presented as means ± SEM. Scale bar 250 µm. * p < 0.05 and **p < 0.01

In order to characterize this non-induced TDTO expression as sign of unstimulated background recombination (leakage), we compared mice of different age, sex and genotype. In mice younger than 15 weeks, homozygous individuals showed a high number of TDTO expressing adipocytes compared to heterozygous ones (18% vs. 70% for females and 9% vs 63% for males; Figure 2(a)). Female mice also showed significantly more non-induced expression of TDTO than respective males in young and old mice (Figure 2(a,b)). Additionally, the number of TDTO expressing adipocytes increased with age of Adipoq-CreER^T2^xTDTO mice (Figure 2(b)). Linear regression showed a significant increase of Cre leakage with age of examined mice independent of sex. Noteworthy, some individual heterozygote female mice also exhibit up to 60% of Cre leakage in older ages (Figure 2(b)).

In summary, all three examined factors impact on the frequency of non-induced TDTO expression. Genotype having the biggest influence with homozygous animals showing more than 60% TDTO expressing adipocytes in each condition without TAM stimulation in Adipoq-CreER^T2^xTDTO reporter mice.

## Discussion

Obesity is linked to numerous diseases, most prominently to diabetes mellitus type 2 [6]. Hence, AT biology, adipocyte lipid metabolism and function of adipokines becoming a more prominent field of interest in obesity and diabetes research. Thus, several AT specific knockout models have been created *in vivo* using the Cre/loxP system after identifying the aP2 promoter in 2003, with this method still being frequently used for targeting AT [7,10].

We aimed at characterizing Cre-mediated gene targeting in the most commonly used model for inducible gene editing in AT, AdipoqCreER^T2^ mice, by visualization the site of recombination *in vivo*. Importantly, AdipoqCreER^T2^ mice show an almost complete and specific Cre catalysed DNA recombination in 97–99% of adipocytes in white AT [9], when challenged by daily TAM injection over 5 days and sacrificed 7 days after last injection. In our *ex vivo* model, we first studied the timeline for TAM induced TDTO expression on a single cell resolution using a live-imaging approach. Noteworthy, TAM induced gene editing, shown by TDTO expression, started 24 h after adding TAM to culture medium (data not shown), plateauing 3 days after application. Importantly, no ectopic TDTO expression was noted in other cells than adipocytes. Next, 1 µM of TAM in culture medium was sufficient to reach Cre catalysed DNA recombination in AT explants, comparable to the results by Sassmann et al. *in vivo* [9]. Of note, 10 µM TAM did not result in faster or stronger Cre induction. Importantly, we could not observe any TAM induced adipocyte browning or adipocyte death *ex vivo*, as suggested by data from *in vivo* TAM administration [11,12]. Hence, these effects may need intact vasculature or nerval innervation of AT as in living animals and seem to be secondary to TAM effects on superior organs, such as the brain or adrenal glands. However, a substantial number of adipocytes also exhibited TDTO expression without TAM application. The number of cells expressing TDTO did not noticeably increase over cultivation period when not challenged by TAM. Thus, the ER^T2^ mediated Cre/loxP system is well suited for gene targeting in adipose tissue, albeit an unstimulated Cre activity (Cre leakage) needs to be considered.

Cre leakage is a known problem of the Cre-ER^T2^ fusion protein regardless of used promoters [16]. The used AdipoqCreER^T2^ mice, however, did not show leakage in LacZ detection of AT [9]. Our live-imaging setup enables detection of TDTO expression following Cre catalysed DNA recombination in living AT on single-cell resolution. Thereby also revealing some Cre leakage in young male mice due to its superior specificity. By quantification and statistical analyses of the non-induced TDTO expression in reporter mice, age, sex and genotype of mice were identified as powerful factors impacting on Cre leakage.

Published studies often use young (<12 weeks) mice for Cre induction [9,16]. Our findings confirm little Cre leakage, especially in young, male mice, of below 10%. With ageing of the animals, however, Cre leakage increased. Young female mice show significantly more non-induced TDTO expression than young male mice and the steeper slope of the linear regression also reveals that Cre leakage increases faster with age in female mice, than in male mice, leading to a significantly higher Cre leakage in old female mice as well. Importantly, the variation found in old, heterozygous females are enormous, leading to non-induced Cre recombination in over 60% of adipocytes in some older female individuals (comparable to young, homozygous mice), which drastically reduces the number of adipocytes left for stimulation. Although the Cre fusion protein appears to be resistant to stimulation by natural ligands of the oestrogen receptor and only susceptible for stimulation by artificial TAM [4], our findings suggest that oestrogen (or one of its natural precursors) have at least some remaining potential to induce translocation of the Cre, thus enabling DNA recombination. Increased levels of estragon in female mice could, therefore, lead to the higher Cre leakage in female mice.

The influence of age is even more significant analysing female and male mice together. This increase with age results from the fact that Cre catalysed DNA recombination being permanent for the individual cell and all descending cells [18]. Therefore, one incident of non-induced Cre activity leads to continuous TDTO expression. Our analysis indicates that in less than 2 weeks such non-induced Cre activity appears in 1% of adipocytes in female mice, while it takes almost 4 weeks for 1% of adipocytes in male mice. However, linear regression is a compromise. For this type of growth, an exponential fit would be more exact, but with the limited number of data points, we feel that a linear fit is a reasonable, more understandable approximation.

The far more drastic impact on Cre leakage, however, has the genotype. Young, homozygous mice showed significantly more non-induced TDTO expression than comparable heterozygous ones. Cre leakage in both, male and female homozygous mice was detected in more than 60% of adipocytes, leaving less than 40% of adipocytes for stimulated induction.

Though Cre leakage has been frequently reported before, it has yet to be fully characterized. We here present three attributes influencing non-induced DNA recombination. To the best of our knowledge, our data represent the first detailed analysis of CreER^T2^ leakage in live mice, showing that this unwanted background recombination in AdipoqCre-ER^T2^ – mediated knockout approaches is sex-, age- and genotype-dependent. When using the Cre-ER^T2^ system we therefore suggest evaluating knockout efficiencies under experiment-equivalent conditions. Importantly, for the success of induced conditional knockout approaches, we strongly advice against using homozygote mice at all.
